# Genome-Wide Association Study of Body Weight in Chicken F2 Resource Population

**DOI:** 10.1371/journal.pone.0021872

**Published:** 2011-07-14

**Authors:** Xiaorong Gu, Chungang Feng, Li Ma, Chi Song, Yanqiang Wang, Yang Da, Huifang Li, Kuanwei Chen, Shaohui Ye, Changrong Ge, Xiaoxiang Hu, Ning Li

**Affiliations:** 1 State Key Laboratory for Agro-Biotechnology, China Agricultural University, Beijing, People's Republic of China; 2 Department of Animal Science, University of Minnesota, Saint Paul, Minnesota, United States of America; 3 Institute of Poultry Science, Chinese Academy of Agricultural Sciences, Yangzhou, People's Republic of China; 4 College of Animal Science and Technology, Yunnan Agricultural University, Kunming, People's Republic of China; Aarhus University, Denmark

## Abstract

Chicken body weight is an economically important trait and great genetic progress has been accomplished in genetic selective for body weight. To identify genes and chromosome regions associated with body weight, we performed a genome-wide association study using the chicken 60 k SNP panel in a chicken F2 resource population derived from the cross between Silky Fowl and White Plymouth Rock. A total of 26 SNP effects involving 9 different SNP markers reached 5% Bonferroni genome-wide significance. A chicken chromosome 4 (GGA4) region approximately 8.6 Mb in length (71.6–80.2 Mb) had a large number of significant SNP effects for late growth during weeks 7–12. The *LIM domain-binding factor 2* (*LDB2*) gene in this region had the strongest association with body weight for weeks 7–12 and with average daily gain for weeks 6–12. This GGA4 region was previously reported to contain body weight QTL. GGA1 and GGA18 had three SNP effects on body weight with genome-wide significance. Some of the SNP effects with the significance of “suggestive linkage” overlapped with previously reported results.

## Introduction

Body weight is an economically important trait for broiler chickens. The identification of DNA polymorphisms and causative genes affecting body weight provides necessary molecular information for marker assisted selection and gene based selection to improve quantitative traits [1], [2]. Several studies reported QTL effects of chicken body weight traits [3], [4], [5], [6], [7], [8]. Many of the QTL results previously reported [9] were from F2 resource populations derived from the cross between parental lines with divergent phenotypic performances. In spite of the existence of previous QTL reports, replication and confirmation of QTL effects are needed, and identifying the exact QTL locations is still a challenge. Most of these reported QTLs for body weight were detected using microsatellite markers with low map resolution, and few causative genes have been identified. The currently available chicken 60 k SNP panel provides genome coverage and map resolution unavailable from microsatellite markers and has the potential of much improved accuracy in finding the exact QTL locations. A recent study [10] showed that designed populations such as F2 populations for genome-wide association studies (GWAS) were advantageous over random populations in reducing false discovery rate (FDR) and in improving mapping accuracy. In this article, we report results of a genome-wide association analysis of chicken body weight using the chicken 60 k SNP panel in a chicken F2 resource population derived from the cross between Silky Fowl and White Plymouth Rock, which are two chicken breeds with highly divergent phenotypes in growth rate and body weight.

## Materials and Methods

### Ethics Statement

Blood samples of chickens were collected from the brachial vein by standard venipuncture procedure #XK622, approved by the Animal Welfare Committee of China Agricultural University.

### Study Population

The study population was the China Agricultural University chicken F2 resource population that was produced from reciprocal crosses of Silky Fowl and White Plymouth Rock which consisted of four half-sibling pedigrees. In this study, 278 individuals of three generations were included. Body weights of the 229 F2 animals were measured weekly from birth to 12 weeks of age, average daily weight gains (ADG) were calculated from birth to 6 weeks of age (ADG6) and from 6 weeks to 12 weeks of age (ADG12). Basic statistics of phenotype data are displayed in Table 1.

**Table 1:** Basic statistics of phenotype data.

Phenotype	Mean	Standard deviation	Minimum	Maximum
BW0^1^	30.2	3.2	22	43
BW1	66.6	13.2	31	97
BW2	138.3	27.0	73	217
BW3	231.7	39.8	106	330
BW4	350.3	60.2	157	506
BW5	506.8	95.7	250	806
BW6	680.1	119.5	344	994
BW7	864.1	163.8	402	1346
BW8	1063.2	194.2	528	1660
BW9	1262.2	221.5	696	1866
BW10	1439.1	256.5	834	2276
BW11	1581.4	284.8	934	2592
BW12	1706.1	308.9	1037	2950
ADG6^2^	15.47	2.82	7.5	22.9
ADG12	24.43	5.71	13.8	47.0

1 The unit of body weight (BW) is gram.

2 The unit of average daily weight gain (ADG) is gram per day.

### Genotyping

Genomic DNA extraction from blood was performed with phenol/chloroform method, and DNA concentration was diluted to 50 ng/ul. The quality and concentration of genomic DNA fulfilled the requirements for the Illumina Infinium SNP genotyping platform. Genotyping using the Illumina 60 K Chicken SNP Beadchip was carried out at the Illumina-certified service provider, DNA LandMarks Inc., Canada. Quality control was assessed in GenomeStudio v2008.1 [11]. One sample was excluded due to low call rate (<95%), and 14,997 SNPs were removed for failing to meet one or more of the following requirements: low call frequency (<95%), low heterozygosity cluster intensity and separation value (<0.4), inheritance or replication error, and low minor allele frequency (<0.1). The final SNP set included 42,639 SNPs for genome-wide association analysis. The marker information on each chromosome is summarized in Table 2.

**Table 2:** Basic information of SNP markers on physical map in chicken.

Chromosome	Physical Map (Mb)	No. of SNP Markers	Marker Density (Kb/SNP)
1	199.4	6,654	30
2	154.4	5,024	30.7
3	113.6	3,849	29.5
4	94	3,120	30.1
5	62	2,025	30.6
6	37.4	1,581	23.7
7	38.4	1,681	22.8
8	30.5	1,320	23.1
9	25.4	1,106	23
10	22.4	1,195	18.7
11	21.9	1,156	18.9
12	20.4	1,275	16
13	18.4	1,080	17
14	15.8	925	17.1
15	13	950	13.7
16	0.43	14	30.7
17	11.2	802	14.0
18	10.9	774	14.1
19	9.8	767	12.8
20	13.9	1,349	10.3
21	6.7	728	9.2
22	3.8	281	13.5
23	6	546	11.0
25	2	150	13.3
24	6.4	671	9.5
26	5.1	588	8.7
27	4.6	427	10.8
28	4.4	510	8.6
E22C19W28_E50C23	0.89	138	6.4
E64	0.049	16	3.1
Z	74.6	1,937	38.5
*Total*	1027.8	42,639	24.1

### Statistical Analysis

Pairwise linkage disequilibrium (LD) measured by r2 values for the F2 population and the parental breeds (12 individuals of White Plymouth Rock and 19 individuals of Silky Fowl) were calculated for each chromosome using PLINK (v1.07) [12].

We assessed the F2 population structure using MDS analysis available from the PLINK software. All autosomal SNPs were pruned using the indep-pairwise option, with a window size of 25 SNPs, a step of 5 SNPs, and r2 threshold of 0.2 [13], resulting in 10,507 independent SNP markers. Pairwise identity-by-state (IBS) distances were calculated between all individuals using the 10,507 independent SNP markers, and MDS components were obtained using the mds-plot option based on the IBS matrix.

Genome-wide association analyses were carried out in PLINK. Linear regression analyses for body weights were performed with the first MDS component, sex, batch, and birth weight as covariates. While the statistical model for ADGs included the first MDS component, sex, and batch as covariates. Measures of SNP effects were calculated by the EPISNP2 package (v3.4) [14]. The fraction of the phenotypic variance explained by the associated SNPs was calculated as previously described [15].

The threshold P-value of the 5% Bonferroni genome-wide significance was calculated based on the estimated number of independent markers and LD blocks for autosome markers [16]. LD block was defined as a set of contiguous SNPs having pairwise r^2^ values exceeding 0.40. Using this approach, the estimated number of independent SNP markers and LD blocks was 25,941, so that the threshold P-value of the 5% Bonferroni genome-wide significance was 1.92×10^−6^ (0.05/25941). The threshold P-value for the significance of “suggestive linkage” that allows one false positive effect in a genome-wide test [17] was calculated using the same approach as above and was 3.85×10^−5^ (1/25941). Empirical genome-wide P-values were obtained from 25,000 permutations for each SNP using the maxT function in PLINK.

## Results and Discussion

### Sample Structure

**Figure 1:**
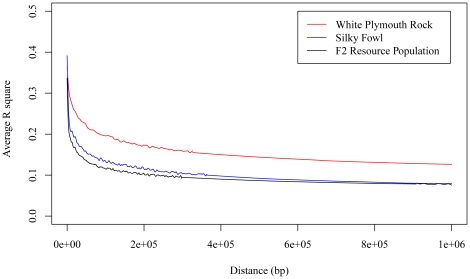
Genome-wide LD pattern of the parental breeds and the whole resource population.

**Figure 2:**
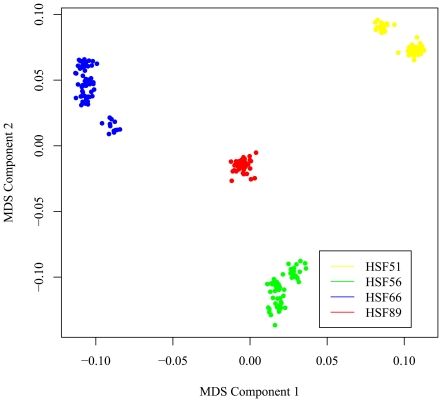
Sample structure identified by multidimensional scaling analysis. HSF is the abbreviation of half-sibling family.

Genome-wide LD pattern of the parental breeds and the whole resource population were analyzed (Figure 1). The White Plymouth Rock had stronger LD than the Silky breed and the F2 offspring, likely due to the fact that White Plymouth Rock has been under intense selection for body weight and growth rate. MDS analysis of 10,507 SNPs with r^2^<0.2 using the first two MDS components (Figure 2) showed that individuals within each half-sib family were clustered together. The first MDS component was used as a covariable to account for sample stratification in the statistical model for testing SNP effects on growth traits as suggested in [18].

### Genome Wide Association Analysis

The global view of P-values for all SNP markers of each trait by a Manhattan plot (Figure S1) using the “gap” package [19] in R v2.12.0 (www.r-project.org) showed that a chicken (*Gallus gallus*) chromosome 4 (GGA4) region was strongly associated with body weight for weeks 7–12 and with average daily gain for weeks 6–12. A total of 26 SNP effects involving 9 different SNP markers reached 5% Bonferroni genome-wide significance under the LD conditions (P<1.92×10^−6^,), and 19 of these 26 SNP effects reached 5% empirical genome-wide significance from permutation tests (Table 3). Of the 19 SNP effects with 5% empirical genome-wide significance, 16 were on GGA4, 2 on GGA1 and 1 one GGA18 (Table 3). The GGA4 region with a - large number of significant SNP effects is a 8.6 Mb region spanning 71.6–80.2 Mb. Recently, the GGA4 region between 60 and 80 Mb on GGA4 was reported to be subjected to recent and ongoing selection in chicken lines with divergent selection on body weight for up to 50 generations using the same 60 K SNP chip [20].

**Table 3:** Genome-wise 5% significant SNPs for body weight traits.

Trait	SNP ID	GGA	Pos (bp) 1	Nearest Gene	SNP	FA^2^	FAW	FAR	FAS	P_value	P_adj^3^	Effect (%)^5^	R2
BW2	GGaluGA118136	18	1356207	*SHISA6*	A/G	A	0.455	0.42	0.42	8.94E-07	0.01828	5.13	0.03
BW7	GGaluGA266058	4	79194441	*LDB2*	A/G	A	0.458	0.54	0.42	5.20E-07	0.022	7.44	0.08
BW8	GGaluGA266058	4	79194441	*LDB2*	A/G	A	0.458	0.54	0.42	1.18E-07	0.00616	7.58	0.09
BW8	Gga_rs15618356	4	77546388	*KCNIP4*	A/G	G	0.676	0.54	0.00	1.33E-06	0.05036	5.94	0.05
BW9	GGaluGA266058	4	79194441	*LDB2*	A/G	A	0.458	0.54	0.42	3.86E-08	0.00156	7.23	0.08
BW10	GGaluGA266058	4	79194441	*LDB2*	A/G	A	0.458	0.54	0.42	8.41E-08	0.00336	7.16	0.08
BW10	Gga_rs15620544	4	79605389	*FBXL5*	A/G	A	0.635	0.58	0.22	1.29E-06	0.04092	6.15	0.06
BW10	Gga_rs16438236	4	80234478	87kb U *BOD1L* 4	A/G	G	0.417	0.63	0.08	1.71E-06	0.05324	5.92	0.05
BW11	GGaluGA266058	4	79194441	*LDB2*	A/G	A	0.458	0.54	0.42	2.76E-07	0.0078	6.64	0.07
BW11	Gga_rs16434462	4	74867005	137 kb U *LOC769270*	A/G	G	0.6	0.79	0.13	4.91E-07	0.01364	5.75	0.05
BW11	Gga_rs14489341	4	74649803	75 kb D *LOC769270* 4	A/G	G	0.6	0.79	0.18	5.98E-07	0.01712	5.98	0.05
BW11	Gga_rs13939265	1	134871532	*OCA2*	A/G	A	0.551	0.71	0.63	1.09E-06	0.03064	5.39	0.04
BW11	Gga_rs16438236	4	80234478	87 kb U *BOD1L*	A/G	G	0.417	0.63	0.08	1.92E-06	0.04992	5.75	0.05
BW12	GGaluGA266058	4	79194441	*LDB2*	A/G	A	0.458	0.54	0.42	6.65E-08	0.00408	7.15	0.08
BW12	Gga_rs16434462	4	74867005	137 kb U *LOC769270*	A/G	G	0.6	0.79	0.13	1.66E-07	0.00888	5.92	0.05
BW12	Gga_rs14489341	4	74649803	75 kb D *LOC769270*	A/G	G	0.6	0.79	0.18	2.24E-07	0.01188	6.15	0.06
BW12	Gga_rs16438236	4	80234478	87 kb U *BOD1L*	A/G	G	0.417	0.63	0.08	4.23E-07	0.02132	5.98	0.05
BW12	Gga_rs13939265	1	134871532	*OCA2*	A/G	A	0.551	0.71	0.63	8.59E-07	0.04188	5.59	0.05
BW12	Gga_rs16432721	4	71673191	92 kb D *TBC1D1*	A/G	A	0.163	0.29	0.08	1.10E-06	0.05208	7.56	0.05
BW12	Gga_rs16434767	4	75515040	28 kb U *STIM2*	A/G	G	0.295	0.46	0.29	1.25E-06	0.0574	5.72	0.04
BW12	Gga_rs15618356	4	77546388	*KCNIP4*	A/G	G	0.676	0.54	0.00	1.90E-06	0.08008	5.82	0.05
ADG12	GGaluGA266058	4	79194441	*LDB2*	A/G	A	0.458	0.54	0.42	4.03E-07	0.02356	7.74	0.05
ADG12	Gga_rs16434462	4	74867005	137 kb U *LOC769270*	A/G	G	0.6	0.79	0.13	4.89E-07	0.02768	6.34	0.04
ADG12	Gga_rs14489341	4	74649803	75 kb D *LOC769270*	A/G	G	0.6	0.79	0.18	5.46E-07	0.03036	6.59	0.04
ADG12	Gga_rs15618356	4	77546388	*KCNIP4*	A/G	G	0.676	0.54	0.00	1.35E-06	0.06848	7.08	0.04
ADG12	Gga_rs16438236	4	80234478	87 kb U *BOD1L*	A/G	G	0.417	0.63	0.08	1.36E-06	0.0686	6.84	0.04

1 Position based on chicken genome build WASHUC2, ‘GGA’ = chromosome of *Gallus gallus*.

2 ‘FA’ = favorable allele, ‘FAW’ =  favorable allele frequency of whole resource population, ‘FAR’ =  favorable allele frequency of F0 White Plymouth Rock, ‘FAS’ =  favorable allele frequency of F0 Silkie Fowl.

3 P_adj indicates p-value adjusted by permutation.

4 ‘U’ =  upstream of, ‘D’  =  downstream of.

5 All the SNP effects are additive.

The *A* allele of GGaluGA266058 within the *LIM domain-binding factor 2* (*LDB2*) gene had the strongest association with late growth (body weights from 7 to 12 weeks of age and ADG12 from 6 to 12 weeks of age). *LDB2* is capable of binding to a variety of transcription factors, and is of vital importance during brain development and blood vessel formation [21], [22]. A polymorphism (Gga_rs16432721) positioned 92 kb downstream of the *TBC1D1* gene was highly significant for body weight at 12 weeks of age. *TBC1D1* was reported to be a candidate gene for obesity in humans [23]. Whole-genome resequencing of several domestic chickens reveals that a mutant *TBC1D1* haplotype has been under selection during domestication in broiler chickens [24]. Several SNPs near *LOC769270* gene had strong association with late growth (body weights from 11 to 12 weeks of age and ADG12). *LOC769270* is a hypothetical protein coding which was bioinformatically predicted in chicken only.

One SNP on GGA1 in the *oculocutaneous albinism II* (*OCA2*) gene had highly significant effects on body weight in weeks 11–12. The association between *OCA2* and body weight in chicken was the first report in this study but the SNP effect in *OCA2* overlapped with a reported body weight QTL region detected in intercrossed lines involving White Plymouth Rock background [25]. In mice, a pigmentation variant of *OCA2* gene is associated with body weight and body size in mouse [26], indicate that *OCA2* gene could be relevant to growth traits.

For early growth traits, only one SNP (GGaluGA118136) on GGA18 had significant association with body weight at 2 weeks of age. The lack of SNP effects on early growth traits could be due to epistatic interaction that may explain more of the genetic variance of early growth than single gene effects [27].

A total of 128 SNP effects involving 61 different SNP markers reached the significance of suggestive linkage (p-value <3.85×10^−5^) (Table S1). These effects were mainly distributed on GGA1, GGA2, GGA3, GGA11, GGA20, and GGA24, and some of those effects overlapped with QTL regions in previous reports. Although the number of effects with suggestive significance is much larger than those with genome-wide significance, most of these effects were still on late growth traits.

Two SNPs located at 151 and 152.3 Mb on GGA1 had effects on body weight in weeks 11–12 and ADG12. This region harbors *glypican 6* (*GPC6*) gene, *glypican 5* (*GPC5*) gene, and gga-mir-17-92 cluster, and is located within the QTL for bodyweight identified in previous studies using the same F2 population as in this study [7], [8]. We also found identical QTL at 68.1 Mb on GGA2 compared with the same study. The glypican proteins have been implicated in the control of cell division and growth regulation [28], but no genetic association had been reported between these two genes and individual body weight or growth rate prior to our study.

A SNP (Gga_rs14373757) within the *Popeye domain-containing protein 1* (*POPD1*) gene on GGA3 had effects on body weight in weeks 10–12 and ADG12, and a polymorphism (Gga_rs15178951) 31 kb downstream of *BMP7* gene on GGA20 had effects on body weight in weeks 11–12. They overlapped with QTL regions reported by two studies [5], [6] on an F2 intercross between two chicken lines divergently selected for bodyweight.

A previous study [4] found that a microsatellite marker (ADL0210) on GGA11 was associated with gizzard weight and another study showed that gizzard weight and body weight at 38 days in chicken had a moderate correlation (r = 0.35) [29]. In our study, an adjacent SNP (Gga_rs15617158) had effects on body weight in weeks 7–10.

Other previous studies [3], [30] found QTL for bodyweight on GGA2 and GGA24. In this study, a SNP within the gene *DYNC1I1* located at 23.9 Mb on GGA2 was associated with body weight in week 6 and ADG6, and two SNPs both located within the *Opioid-binding protein/cell adhesion molecule-like* (*OPCML*) gene on GGA24 were found to be associated with body weight in week 12 and ADG12. A SNP (Gga_rs14269721) within the gene Cbfa2t2 on GGA20 was in association with body weight in week 12 and ADG12. This is a new QTL identified in this study only.

In summary, our GWAS detected 26 SNPs with genome-wise significance and 128 SNPs with the significance of suggestive linkage. Most of these SNPs were reported for the first time. Many of the SNP effects overlapped with previously reported QTL regions, providing evidence towards confirmation of QTL effects. The results are also helpful for identifying the exact QTL locations because of the much improved map resolution of the 60 k SNP panel over the map resolution of microsatellite markers used by most of previous reports on chicken QTL effects.

## Supporting Information

Figure S1 **Manhattan plot of genome-wide association analysis for body weight traits.** The dashed line indicates genome-wise significance of suggestive association (p-value <3.85×10−5), and the solid line declares genome-wise 5% significance with a p-value threshold of 1.92×10^−6^.
(PDF)

Table S1 **Associated SNP with genome-wise significance of suggestive association for body weight traits.**
(XLS)
