# Supplementary material for: Genome-Wide Association Study of Body Weight in Chicken F2 Resource Population
Source: PLoS One. 2011 Jul 14;6(7):e21872. doi: 10.1371/journal.pone.0021872 (PMC3136483; doi:10.1371/journal.pone.0021872)
Supplement: Figure S1 — Manhattan plot of genome-wide association analysis for body weight traits. The dashed line indicates genome-wise significance of suggestive association (p-value <3.85×10^−5^), and the solid line declares genome-wise 5% significance with a p-value threshold of 1.92×10^−6^. (PDF) [file pone.0021872.s001.pdf]

Manhattan Plot of Genome−wide Association Analysis for BW1

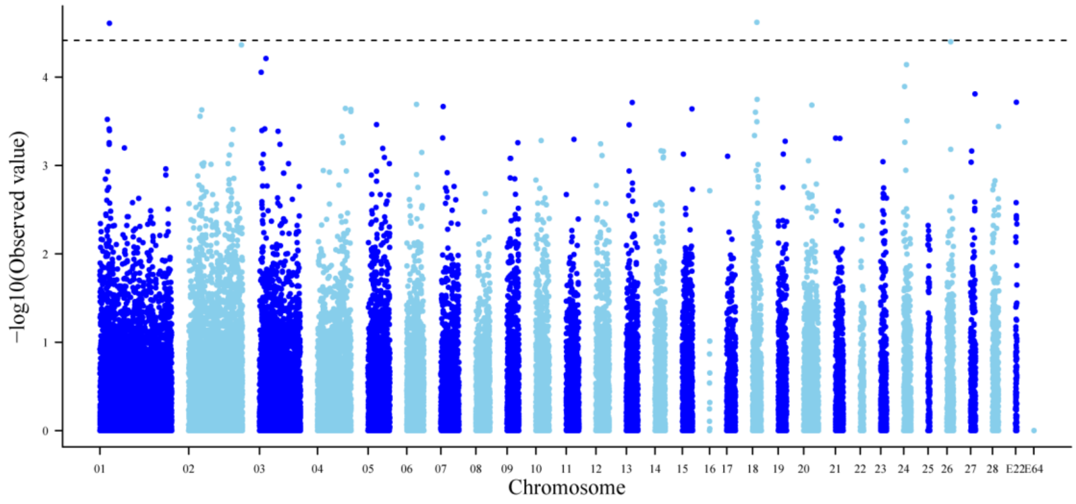

Manhattan Plot of Genome−wide Association Analysis for BW2

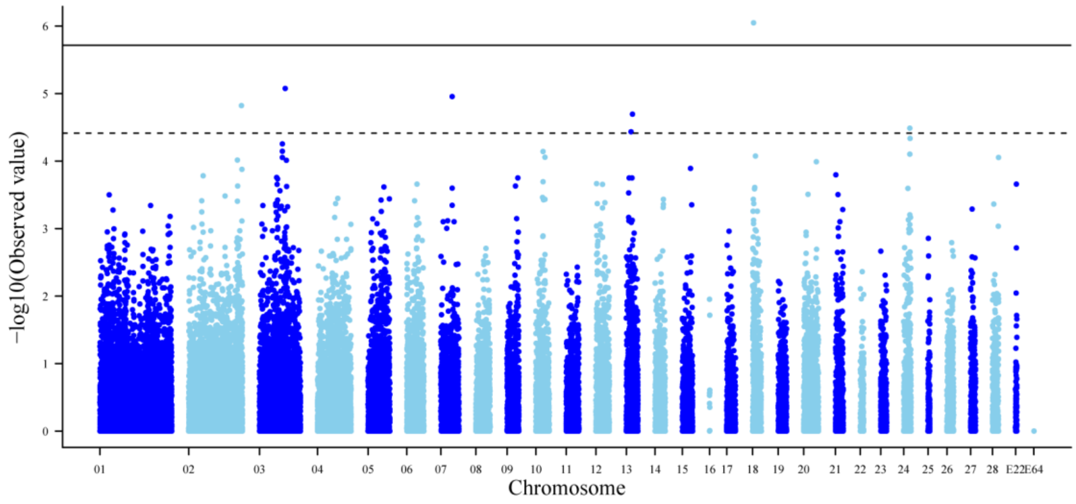

Manhattan Plot of Genome−wide Association Analysis for BW3

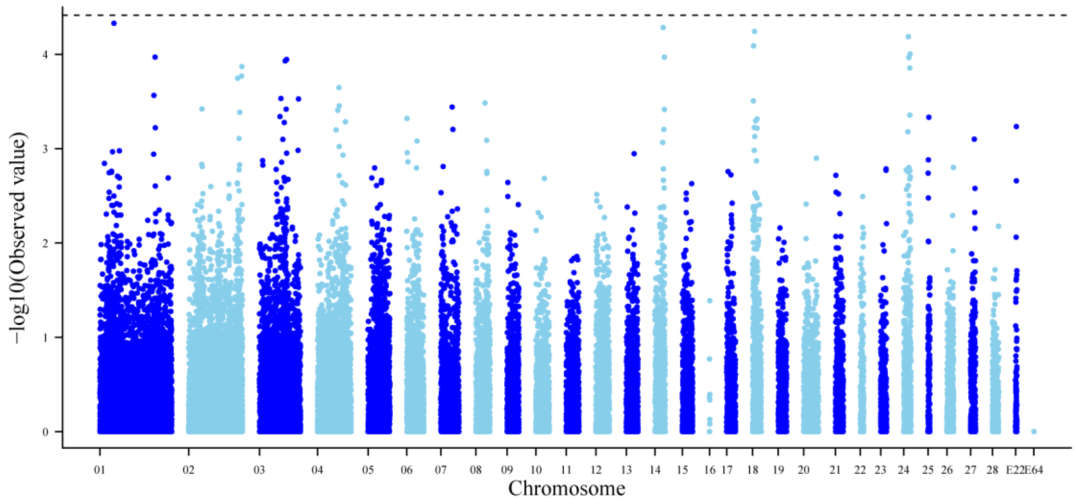

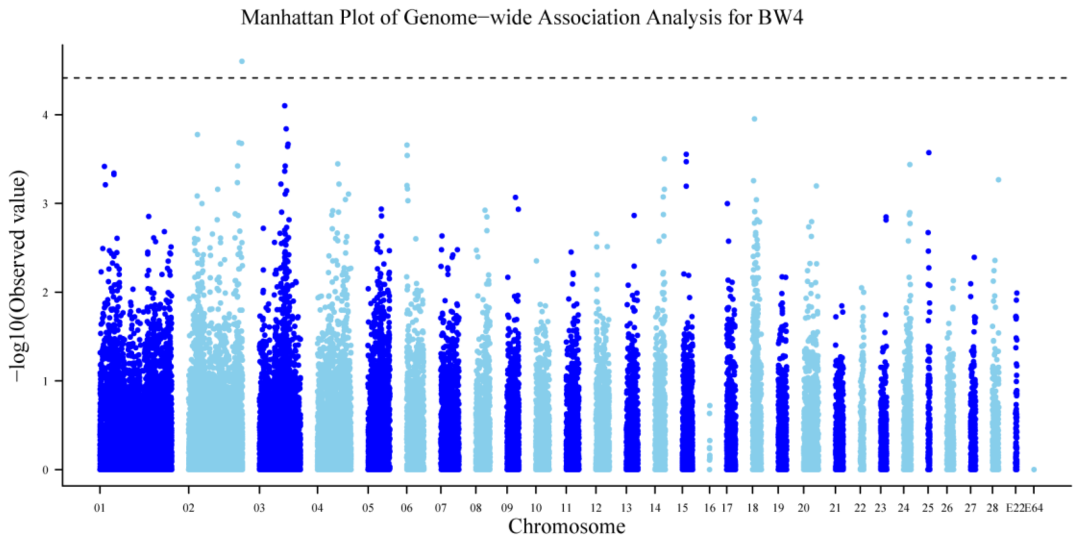
Manhattan Plot of Genome−wide Association Analysis for BW4
−log10(Observed value)
0
1
2
3
4
01 02 03 04 05 06 07 08 09 10 11 12 13 14 15 16 17 18 19 20 21 22 23 24 25 26 27 28 E22E64
Chromosome

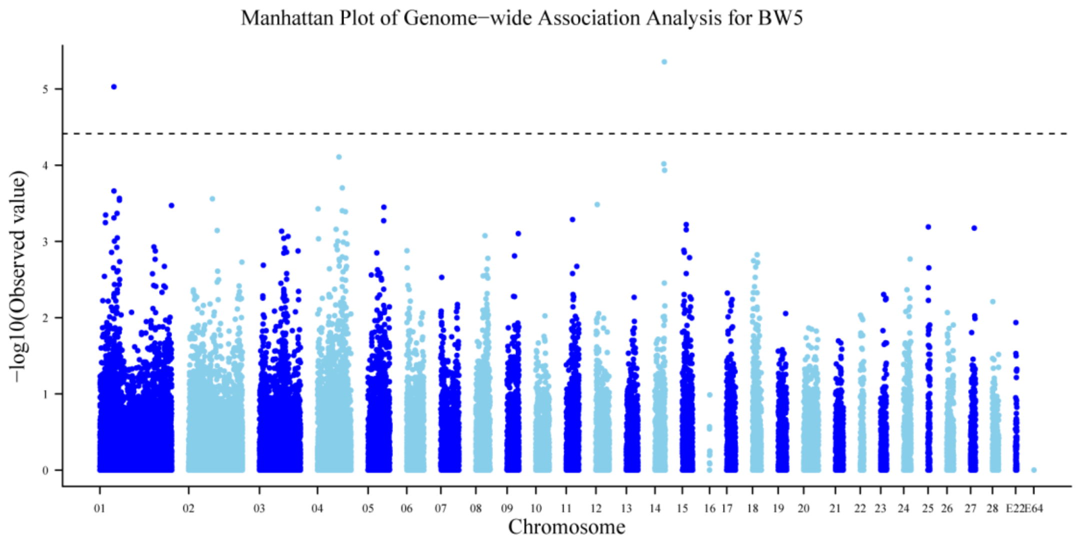
Manhattan Plot of Genome−wide Association Analysis for BW5
−log10(Observed value)
0
1
2
3
4
5
01 02 03 04 05 06 07 08 09 10 11 12 13 14 15 16 17 18 19 20 21 22 23 24 25 26 27 28 E22E64
Chromosome

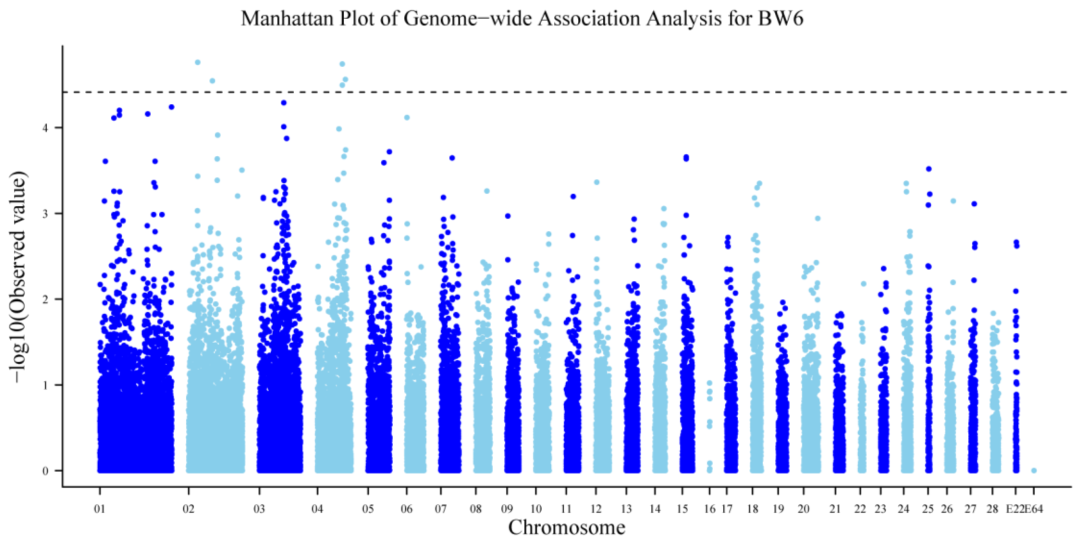
Manhattan Plot of Genome−wide Association Analysis for BW6
−log10(Observed value)
0
1
2
3
4
01 02 03 04 05 06 07 08 09 10 11 12 13 14 15 16 17 18 19 20 21 22 23 24 25 26 27 28 E22E64
Chromosome

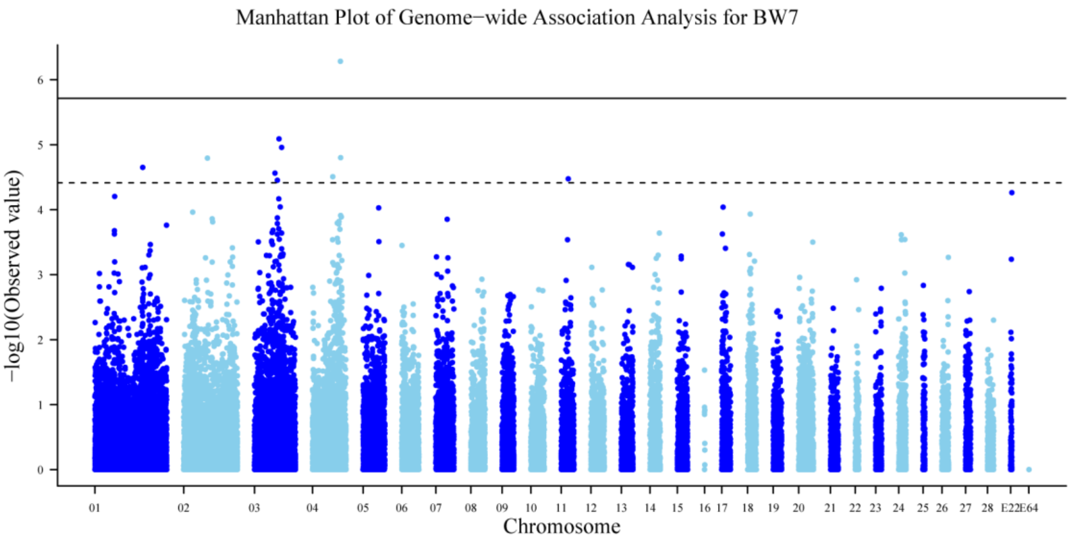
Manhattan Plot of Genome−wide Association Analysis for BW7
−log10(Observed value)
0
1
2
3
4
5
6
01 02 03 04 05 06 07 08 09 10 11 12 13 14 15 16 17 18 19 20 21 22 23 24 25 26 27 28 E22E64
Chromosome

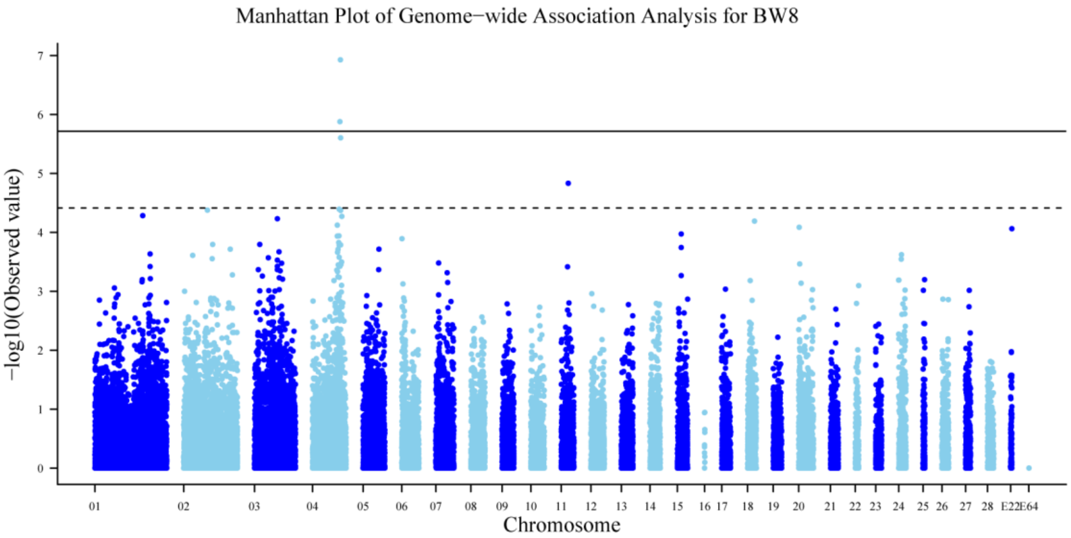
Manhattan Plot of Genome−wide Association Analysis for BW8
−log10(Observed value)
0
1
2
3
4
5
6
7
01 02 03 04 05 06 07 08 09 10 11 12 13 14 15 16 17 18 19 20 21 22 23 24 25 26 27 28 E22E64
Chromosome

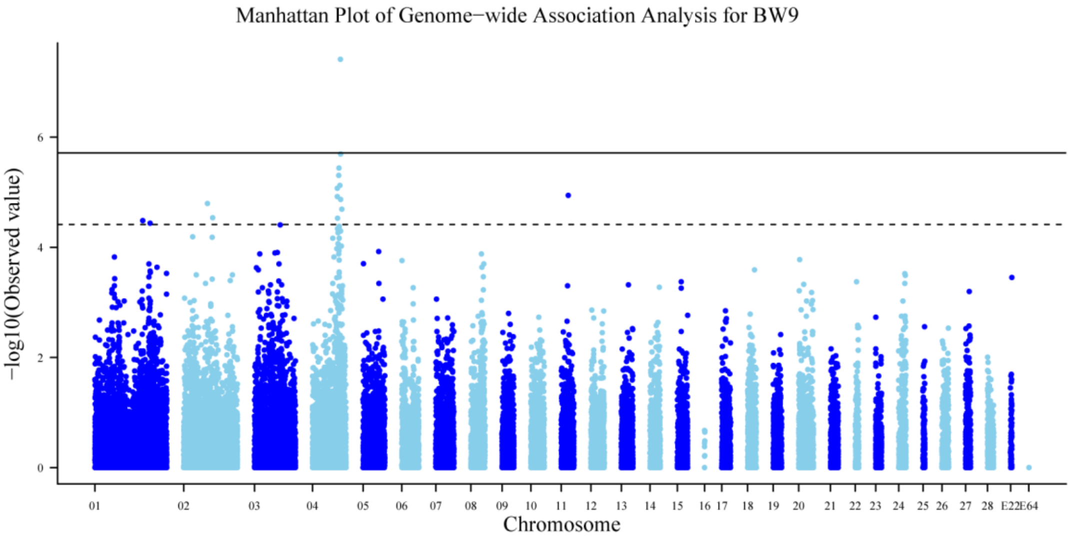
Manhattan Plot of Genome−wide Association Analysis for BW9
−log10(Observed value)
0
2
4
6
01 02 03 04 05 06 07 08 09 10 11 12 13 14 15 16 17 18 19 20 21 22 23 24 25 26 27 28 E22E64
Chromosome

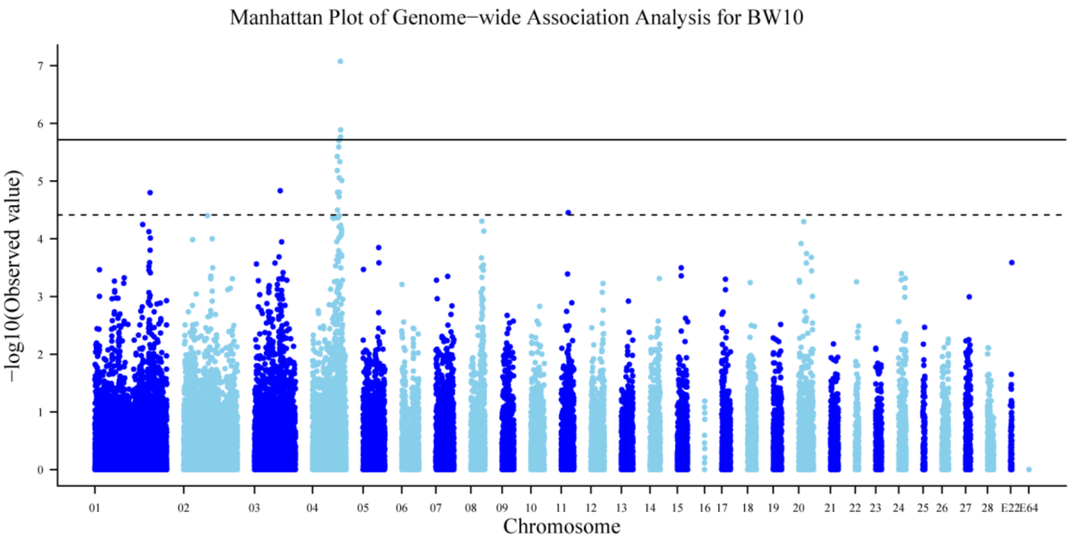
Manhattan Plot of Genome−wide Association Analysis for BW10
−log10(Observed value)
0 1 2 3 4 5 6 7
01 02 03 04 05 06 07 08 09 10 11 12 13 14 15 16 17 18 19 20 21 22 23 24 25 26 27 28 E22E64
Chromosome

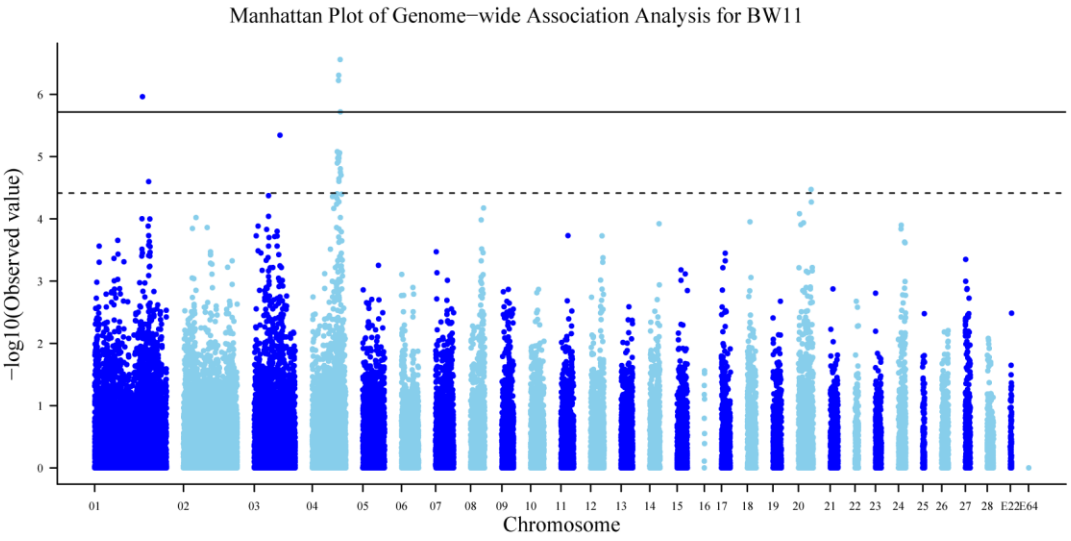
Manhattan Plot of Genome−wide Association Analysis for BW11
−log10(Observed value)
0 1 2 3 4 5 6
01 02 03 04 05 06 07 08 09 10 11 12 13 14 15 16 17 18 19 20 21 22 23 24 25 26 27 28 E22E64
Chromosome

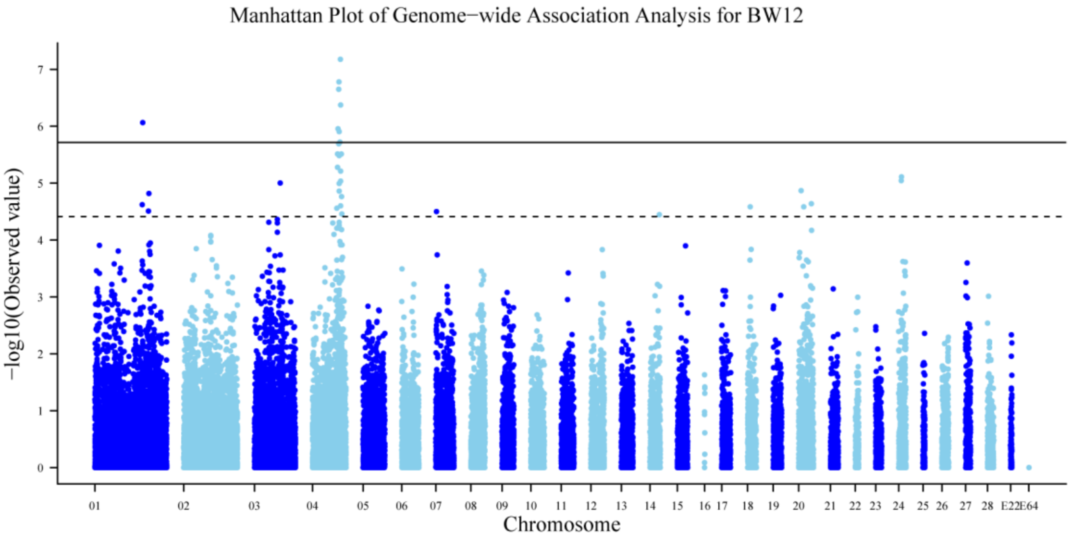
Manhattan Plot of Genome−wide Association Analysis for BW12
−log10(Observed value)
0 1 2 3 4 5 6 7
01 02 03 04 05 06 07 08 09 10 11 12 13 14 15 16 17 18 19 20 21 22 23 24 25 26 27 28 E22E64
Chromosome

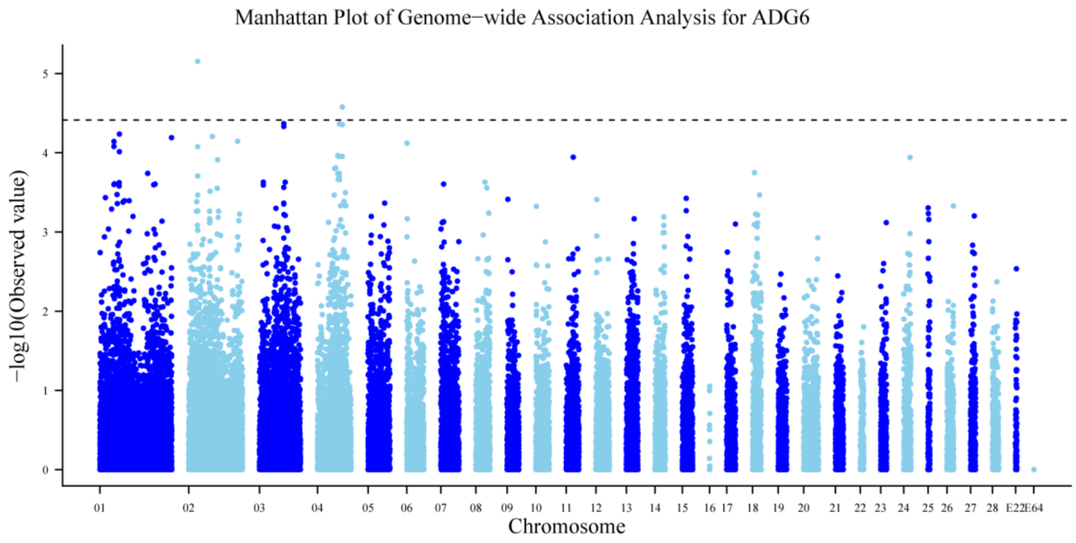

Manhattan Plot of Genome−wide Association Analysis for ADG6
−log10(Observed value)
Chromosome
01
02
03
04
05
06
07
08
09
10
11
12
13
14
15
16
17
18
19
20
21
22
23
24
25
26
27
28
E22
E64

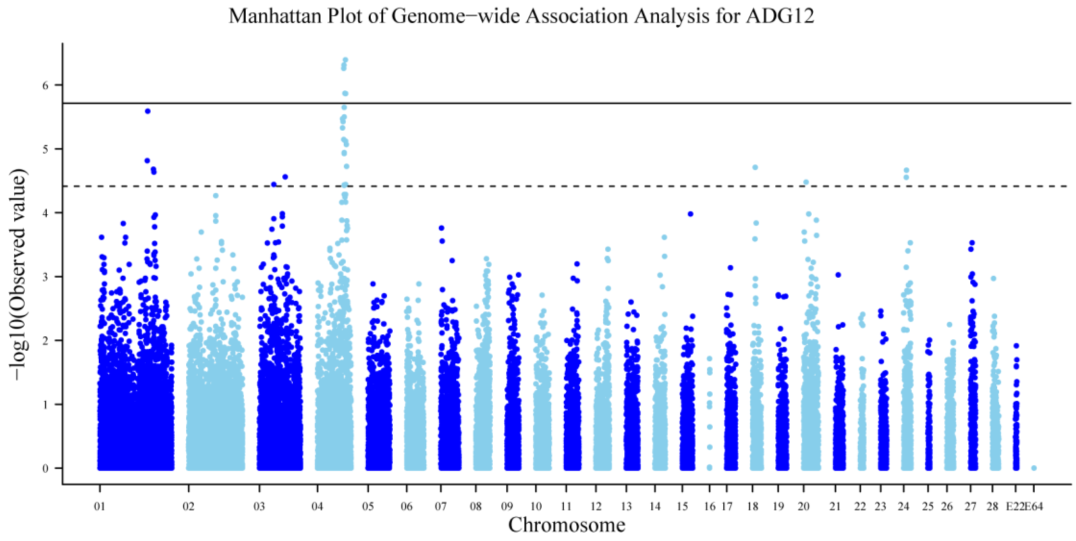

Manhattan Plot of Genome−wide Association Analysis for ADG12
−log10(Observed value)
Chromosome
01
02
03
04
05
06
07
08
09
10
11
12
13
14
15
16
17
18
19
20
21
22
23
24
25
26
27
28
E22
E64
